# *M.marinum* lacking *epsH* shows increased biofilm formation in vitro and boosted antibiotic tolerance in zebrafish

**DOI:** 10.1038/s41522-025-00743-5

**Published:** 2025-06-14

**Authors:** Saara Lehmusvaara, Alina Sillanpää, Milan Wouters, Rosa Korhonen, Nelli Vahvelainen, Hanna Luukinen, Paulina Deptula, Kirsi Savijoki, Milka Hammarén, Mataleena Parikka

**Affiliations:** 1 Faculty of Medicine and Health Technology, Tampere University, Tampere, Finland; 2 Laboratory of Microbiology, Parasitology and Hygiene (LMPH), Faculty of Pharmaceutical, Biomedical and Veterinary Sciences, University of Antwerp, Wilrijk, 2000 Antwerp, Belgium; 3 Department of Food Science, Faculty of Science, University of Copenhagen, Rolighedsvej 26, 1958 Frederiksberg C, Denmark; 4 Faculty of Pharmacy, Division of Pharmaceutical Chemistry and Technology, University of Helsinki, Helsinki, Finland; 5 Faculty of Agriculture and Forestry, FI-00014, University of Helsinki, Helsinki, Finland; 6 European Molecular Biology Laboratory, Heidelberg, Germany

**Keywords:** Antimicrobials, Biofilms, Pathogens

## Abstract

Recent discoveries have indicated that biofilm communities may play a role in natural drug tolerance of *Mycobacterium tuberculosis*. A transposon-based mutation library of a closely related species, *Mycobacterium marinum*, was used to identify clones in which the relative amount of extracellular DNA (eDNA), an important component of the extracellular matrix of biofilms, is altered. The disruption of a putative glycosyl transferase gene QDR78 11175, *epsH*, caused a substantial increase of the eDNA content of biofilms, and increased the growth rate and the biomass/cell in biofilm-forming conditions compared to wild-type. The increased abundance of biomass was mainly due to the elevated levels of eDNA and proteins in the extracellular matrix. The growth of the Δ*epsH* strain in the zebrafish was normal, but the mutant developed greater antibiotic tolerance in the adult zebrafish model. These results suggest that the extracellular matrix of biofilms increases antibiotic tolerance of mycobacteria during infection.

## Introduction

*Mycobacterium tuberculosis* is the causative agent of tuberculosis (TB), which has infected approximately 25% of the world’s population and killed 1.25 million people in 2023^1^. After the COVID-19 pandemic, TB is once again the world’s leading cause of death from a single infectious agent^1^. The WHO-recommended treatment of TB requires the combination of four antibiotics, rifampicin, isoniazid, pyrazinamide, and ethambutol, for 2 months, followed by four months of the first two drugs. Alternatively, a combination of isoniazid, rifapentine, moxifloxacin, and pyrazinamide for four months can be used^2^. This long treatment schedule can be attributed to the tolerant nature of a *M. tuberculosis* infection.

Recently, biofilms have been associated with *M. tuberculosis* infections^3^. Biofilms are defined as aggregated bacterial communities in which cells are embedded in a self-produced matrix of extracellular polymeric substances^4^, often referred to as the extracellular matrix (ECM). Recent findings suggest that the slow growth and the asymmetrical cell division of the bacteria contribute to the antibiotic tolerance of mycobacteria^5–7^. In addition, the ECM of mycobacterial biofilms contributes to virulence and antibiotic tolerance in vivo^3^. Biofilm-like bacterial communities with ECM were detected in lung tissue samples from mice, macaques, and TB patients^3^, and the disintegration of mycobacterial ECM in the mouse model reduced both the virulence and the antibiotic tolerance^3^. In order to be able to develop more efficient therapies against tuberculosis and other mycobacterial infections, better understanding of mycobacterial biofilms is needed.

Similarly to other bacterial biofilms^4,8^, the ECM of cultured mycobacterial biofilms contains eDNA, lipids, proteins, and polysaccharides, especially cellulose^3,9,10^. Extracellular DNA (eDNA) is present in biofilms of various bacterial species, including mycobacteria. Studies with DNase treatment of biofilms have revealed that eDNA is commonly deposited outside the cells in the early stage of biofilm formation, where it aids the bacterial attachment and aggregation^11–14^. In addition, eDNA has been observed to promote controlled cell dispersion^15^, and it was recently found to have an important structural role in the formation of the biofilms of slow-growing mycobacteria. In contrast, eDNA was absent in fast-growing mycobacteria^16^. eDNA is recognized for its ability to inactivate cationic antibiotics, like aminoglycosides, presumably due to electrostatic interactions^17^. Lastly, the enzymatic degradation of the eDNA of mycobacterial biofilms reduced tolerance to other antibiotics^16^, providing further evidence that eDNA has a more general role in antibiotic-tolerant biofilms.

**Fig. 1:**
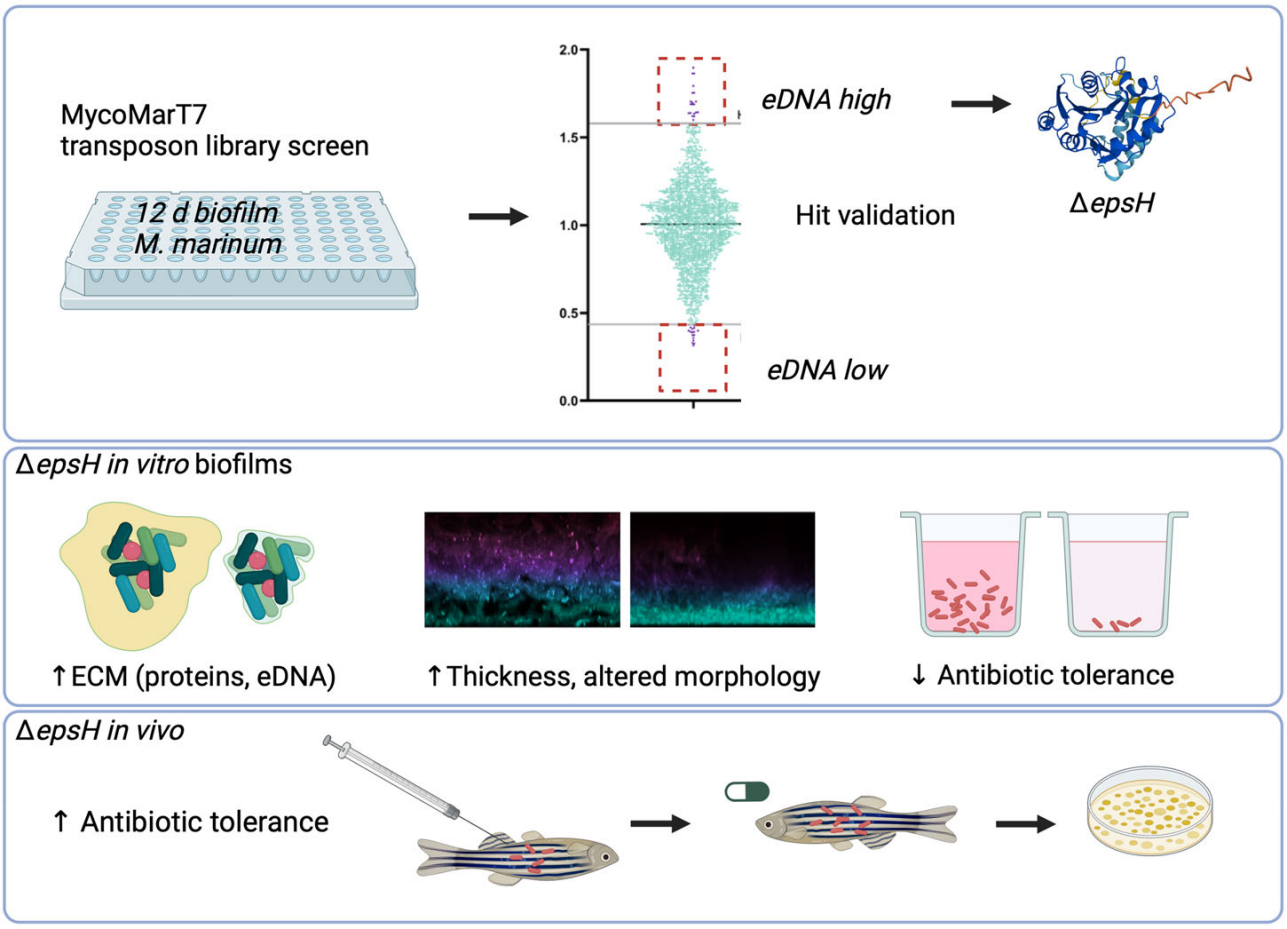
A summarizing figure of the work. A transposon library screen of *Mycobacterium marinum* identified a biofilm mutant that lacked the gene encoding for a putative glycosyl transferase called *epsH*. The mutant had increased production of extracellular matrix in which extracellular DNA and proteins were increased. The drug tolerance of the mutant was decreased in biofilms cultured in vitro but increased in vivo in the adult zebra fish.

Acknowledging the central role of eDNA in slow-growing mycobacterial biofilms and in antibiotic tolerance, we set out to identify mutants with altered eDNA abundance in biofilm-inducing conditions. To achieve this, a transposon-based mutation library of *Mycobacterium marinum* ATCC 927 ^T^, the causative agent of fish tuberculosis, was screened, identifying several mutants with significantly increased and decreased levels of eDNA (Fig. 1). A mutant clone with the highest detected abundance of eDNA was further characterized. Sequencing revealed that a transposon was disrupting *epsH* gene coding for a putative glycosyltransferase EpsH. Disruption of *epsH* resulted in variations in ECM composition, with the mutant producing more ECM per cell than the wild type. In addition to elevated eDNA levels, the mutant also exhibited increased extracellular protein levels. As predicted, the Δ*epsH* strain demonstrated increased antibiotic tolerance in the adult zebrafish model.

## Results

### Transposon library screen identifies *high eDNA* and *low eDNA* mutants in *M. marinum* biofilms

**Fig. 2:**
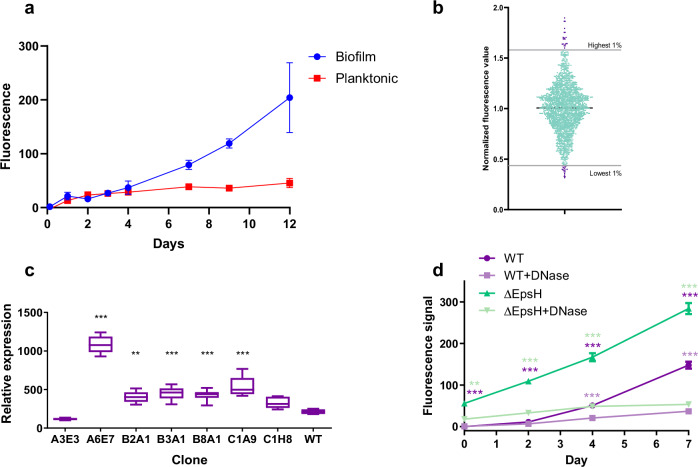
MycoMarT7 transposon library screen reveals mutants with altered eDNA levels in biofilm-forming culture conditions. **a** GelRed-based measurement of eDNA levels in biofilm and planktonic *M. marinum* cultures revealed higher eDNA content in the biofilm. **b** Normalized fluorescence values of single clones grown in biofilm forming conditions after GelRed staining. Values were normalized to the average value of the plate. The clones having highest and lowest 1% of fluorescence were selected for further analysis. **c** Secondary screen of top and bottom 1% of eDNA abundancy clones by AccuBlue staining confirms the altered eDNA levels. One-way ANOVA followed by Dunnett's multiple comparison test. **d** The fluorescence signal of DNA binding dye AccuBlue increases by time in biofilm forming conditions and is significantly higher in *epsH* mutant strain than in WT. DNase treatment significantly reduces the fluorescence signal. The colour of the asterisk indicates the condition which the statistical analysis is compared to. Two-way ANOVA followed by Bonferroni’s multiple comparison test. P-values: * < 0.05, ** < 0.01, *** < 0.001.

Biofilms of *M. marinum* were found to contain significantly higher levels of eDNA compared to planktonic cultures, suggesting that eDNA is a prominent component of the biofilm ECM of this species (Fig. 2a). To identify genes involved in eDNA deposition to mycobacterial biofilms, we created a transposon-based randomly mutated library of *M. marinum* using a *mariner*-based system and phage transduction^18^. 1 939 individual mutants were cultured in biofilm-inducing microaerophilic conditions, and their eDNA levels were measured by cell impermeable GelRed staining (Fig. 2b). To account for the possible defects in growth, the GelRed signal was normalized to the OD_600_ of the respective well. 1% of the clones with the highest (‘*high eDNA’*) or lowest (‘*low eDNA’*) eDNA levels were selected for further analysis, to understand the cellular mechanisms regulating eDNA deposition into biofilm ECM. Unfortunately, despite our efforts only 18% (7/39) of these 39 hit clones were culturable on agar plates. To validate the screening results for the culturable hit clones, eDNA in biofilms was quantified with an alternative method (Fig. 2c). As GelRed binds both DNA and RNA (Supplementary Fig. 1), we used a DNA-specific, non-cell-permeable dye, AccuBlue (Supplementary Fig. 2). AccuBlue-based quantification verified five *high eDNA* -mutants and confirmed that the highest eDNA abundance was detected in the A6E7 mutant biofilms (Fig. 2c). In a longitudinal measurement of this *high eDNA* -clone with AccuBlue we saw that the relative amount of eDNA in ECM increased more rapidly compared to wild type biofilms. The AccuBlue signal could efficiently be abolished by a DNase treatment of the A6E7 and wild-type biofilms, confirming the specificity of the staining (Fig. 2d).

### A mutant lacking a putative glycosyltransferase epsH has the highest level of eDNA under biofilm-forming conditions

To reveal the insertion sites, the whole genomes of three identified clones with the highest abundances of eDNA were sequenced. In the clone with the highest eDNA signal (A6E7), the sequencing revealed a single transposon insertion which disrupted the gene QDR78_11175. Intriguingly, the transposon was inserted into the 5’ end of the gene keeping the first 610 bases out of 750 intact, corresponding to 204 aa of 249 in the product and located downstream of the glycosyltransferase active site, yet the insertion into this distal region of the gene still caused a clearly altered, *high eDNA* phenotype especially under biofilm-forming conditions. Using PROKKA 1.11., this gene was previously annotated (GenBank ID RFZ58776.1) by Das et al. (2018) as a putative glycosyl transferase EpsH based on its similarity to a known protein in *Bacillus subtilis*^19^. The Conserved Domains Database (CDD) search of the EpsH of *M. marinum* also identifies a BcsA domain, which has known functions in the catalytic BcsA-B protein complex responsible for cellulose synthesis in well studied Proteobacteria^20^. However, orthologues of BcsA or BcsB cellulase synthase subunits are not found in the genome of *Mycobacterium tuberculosis* and the cellulose synthesis machinery of mycobacteria is currently unknown^20^.

**Fig. 3:**
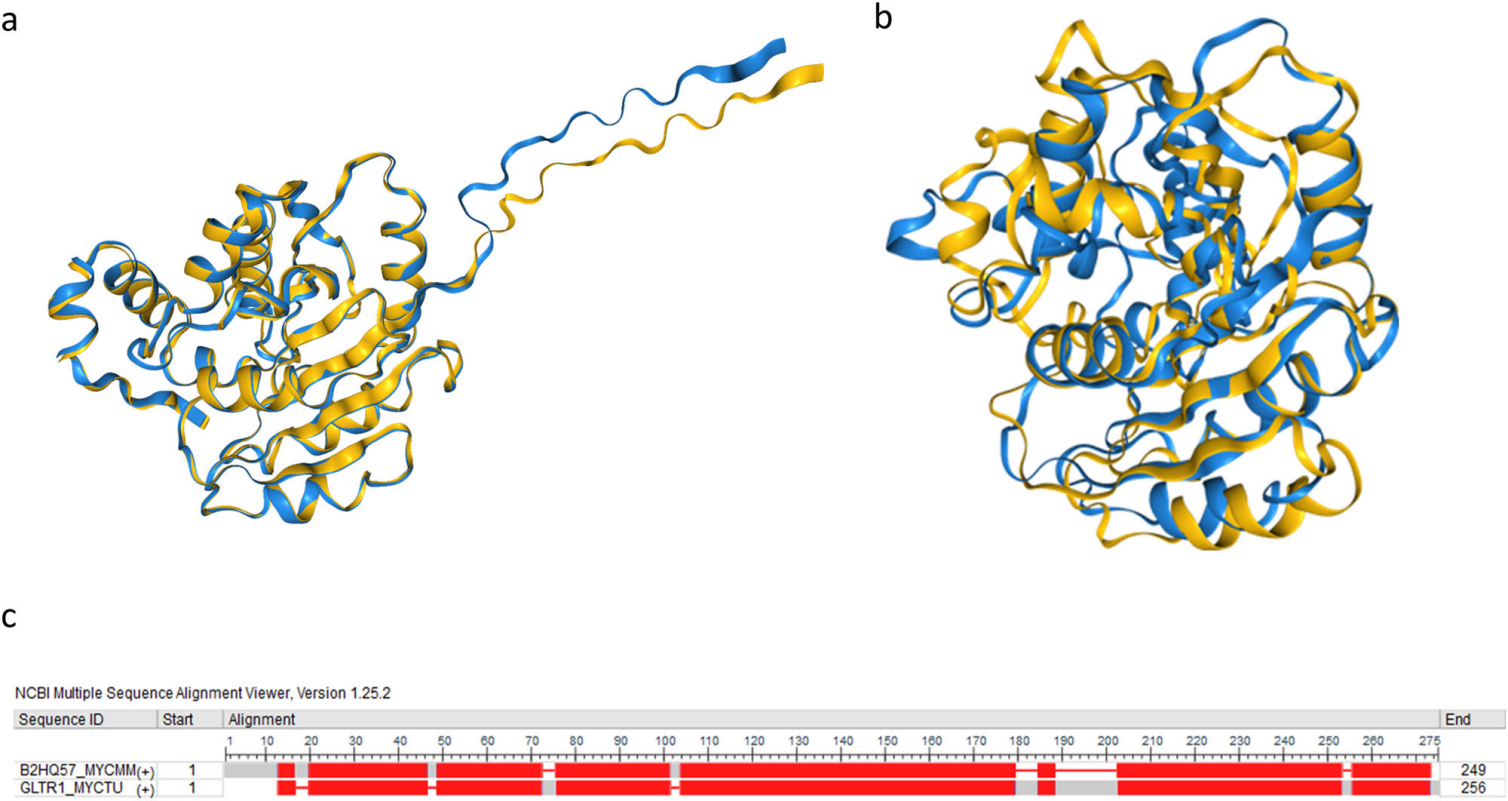
Biofilm screen identifies a high eDNA clone lacking an EpsH glycosyltransferase. Based on sequence homology and BLASTP analysis, *M. ulcerans* also has EpsH in its genome. Figure **a** shows the structural overlap of the structures of *M. ulcerans* EpsH and *M. marinum* EpsH structures as predicted by AlphaFold 2.0. The overlapping image was created with FoldSeek. OrthoInspector 3.0 identified Rv2957 as the closest homologous protein in Mtb. Figure **b** shows the structural overlap of the structures of Rv2957 and *M. marinum* EpsH structures as predicted by AlphaFold 2.0. The overlapping image was created with FoldSeek. **c** The sequence alignment shows the conserved areas between *M. marinum* EpsH and Rv2957 in red.

In *M. ulcerans*, an identical protein to the EpsH of *M. marinum* can be found (Fig. 3a). Based on the OrthoInspector 3.0 analysis^21^, the most similar protein in *M. tuberculosis* is Rv2957, a PGL/p-HBAD biosynthesis glycosyltransferase (Fig. 3b, c). The same protein was suggested to be the closest homologue in *M. tuberculosis* based on AlphaFold and subsequent FoldSeek analysis^22^. Figure 3a, b show the overlap between the predicted structures of *M. marinum* EpsH and the *M. ulcerans* EpsH (a) the *M. tuberculosis* Rv2957 (b), suggesting a close structural relationship.

In the other two sequenced *high eDNA* hit clones (A5A8 and A3E5) the transposon disrupted genes QDR78_00940 coding for a TetR transcriptional regulator (WOR04855.1) and QDR78_22550 coding for a sterol desaturase (WOR03890.1), respectively. CDD search indicated that the TetR family regulators “are involved in the transcriptional control of multidrug efflux pumps, pathways for the biosynthesis of antibiotics, response to osmotic stress and toxic chemicals, control of catabolic pathways, differentiation processes, and pathogenicity may play a role in pathogenicity” pointing to its role in interaction with the environment. On the other hand, sterol desaturase (WOR03890.1) returns a specific hit to ERG3 sterol desaturase and is described as a “Sterol desaturase/sphingolipid hydroxylase, fatty acid hydroxylase superfamily [Lipid transport and metabolism]”, which could have a role in establishing mycobacterial persistency^23^. Interestingly, QDR78_22550 is located directly upstream of a gene coding for another TetR/AcrR transcriptional regulator, which is additionally the only gene in the area encoded on a negative strand. While it would be tempting to speculate that the transposon insertion has an indirect effect on the function of TetR/AcrR transcriptional regulator and therefore interaction with the environment, the significance of this location remains unclear.

Interestingly, our previous surface proteomics study of *M. marinum* biofilms revealed that EpsH is present in the extracellular matrix (ECM) of both early submerged biofilms (two days old) and mature pellicle biofilms (four weeks old). In contrast, EpsH was not detected on the cell surface of planktonic cells^7^. These proteomics results suggest a role for EpsH in the ECM of biofilms at different stages of development.

As the top hit with the highest level of eDNA and due to its possible role in early biofilms as suggested by our previous proteomics analyses, EpsH was chosen as the subject of our follow-up studies.

### Complementation of the Δ*epsH* strain reverses the *high eDNA* phenotype under biofilm-forming conditions

**Fig. 4:**
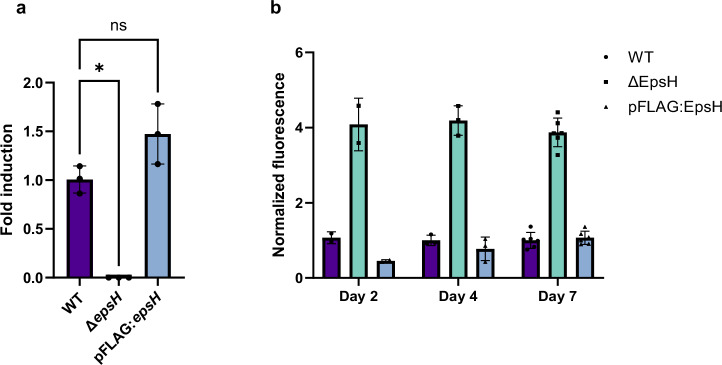
Δ*epsH* has an altered phenotype in eDNA deposition under biofilm-forming conditions. **a** Quantitative PCR analysis of *epsH* expression in WT, Δ*epsH* and pFLAG:*epsH* strain shows restored *epsH* expression in the complemented strain. One-way ANOVA followed by Šídák's multiple comparisons test, error bar: mean ± SD. Three biological replicates were performed and shown as individual points. **b** Accublue staining of the biofilms shows that the complementation of the *epsH* gene reduces the level of eDNA close to the WT. Fluorescence values were normalized to OD_600_. Error bar: mean ± SD.

To confirm that the lack of *epsH* caused the phenotype with increased eDNA deposition into biofilm ECM, we constructed a complemented strain (pFLAG:*epsH*) to restore wild type *epsH* expression in the Δ*epsH* mutant. Quantitative PCR analysis confirmed that *epsH* expression was successfully restored in the complemented strain to levels comparable to WT cells (Fig. 4a). Using AccuBlue staining to quantify eDNA levels, we observed that complementation significantly reduced the amount of eDNA in biofilms compared to the Δ*epsH* mutant, bringing levels close to those of the WT (Fig. 4b). The complementation confirmed that the lack of *epsH* caused the increased eDNA deposition in biofilm-forming conditions.

### The deletion of *epsH* leads to an increased growth rate in biofilm culturing conditions

**Fig. 5:**
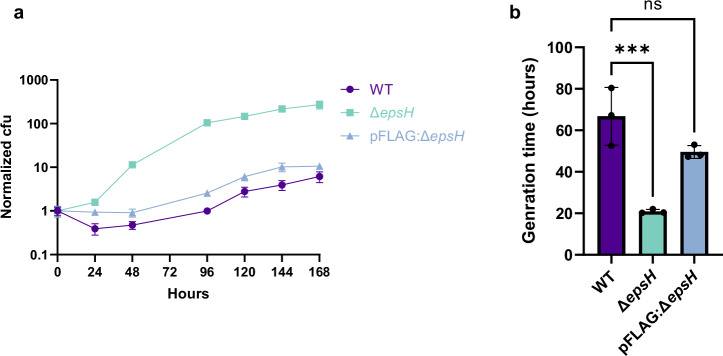
*epsH* deletion affects the growth in biofilm-forming conditions. **a** CFU counts indicate faster cell proliferation of Δ*epsH* in biofilm-forming conditions compared to the WT. **b** Δ*epsH* demonstrated significantly shorter generation time in comparison to WT. One-way ANOVA followed by Dunnett’s multiple comparisons test, error bar: mean ± SD. ***p = 0.0009.

To evaluate the effects of *epsH* deletion, we compared WT and Δ*epsH* strains under planktonic and biofilm conditions. In planktonic culture, compared to wild type, the generation time of the mutant strain was not significantly altered (Supplementary Fig. 3). However, in the biofilm culture conditions in the absence of detergent or glycerol, the growth rate of the Δ*epsH* strain was substantially increased (Fig. 5a, b). These results on growth kinetics of the Δ*epsH* strain suggest that EpsH influences the growth rate of mycobacteria under biofilm growth conditions.

### The deletion of *epsH* increases the abundance of ECM under biofilm-forming conditions

**Fig. 6:**
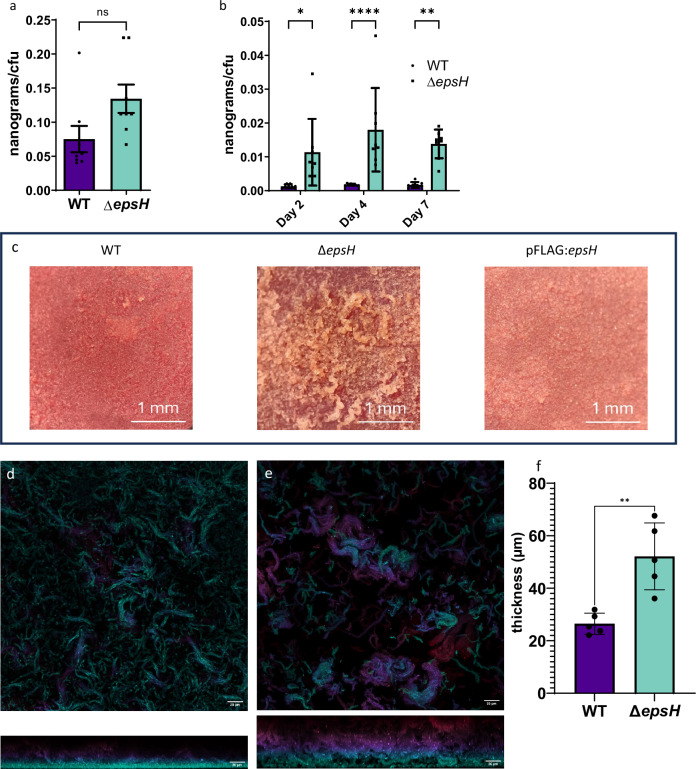
*epsH* deletion increases biomass per cell and affects colony morphology and biofilm structure. **a** *epsH* deletion does not significantly influence the biomass in planktonic condition after 7 days of culture. Unpaired t test, error bar: mean ± SEM. **b** *epsH* deletion increases the total biomass per CFU under biofilm-forming conditions. Two-way ANOVA followed by Šídák's multiple comparisons test. *p = 0.0131, **p = 0.0023, ****p < 0.0001, error bar: mean ± SEM. **c** 2-week-old WT, Δ*epsH* and pFLAG:*epsH* biofilm on Congo red agar. 2-week-old WT and pFLAG:*epsH* bacterial lawns on agar have smoother surface compared to the rough morphology of the Δ*epsH* strain. The images were taken through a stereo microscope using 5x magnification. Confocal microscope images of 7-day-old tdTomato expressing **d** WT and **e** Δ*epsH* biofilms show differences in biofilm structure and thickness. Images were taken with Nikon A1R+ confocal laser scanning microscope equipped with a Nikon Apo LWD 40x WI λS DIC N2 objective. Image processing was carried out with Fiji-ImageJ software. Color represents z-distance, with warmer colors indicating higher positions and cooler colors showing deeper regions of the biofilm. **f** Δ*epsH* biofilms are significantly thicker in comparison to WT biofilms (*N* = 5). Biofilm thickness was calculated from confocal z-stacks acquired with a 0.325 µm step size. Slices with a mean intensity below 15 were excluded from the top and bottom, and the remaining slices were multiplied by the step size to determine total thickness. Unpaired t test with Welch's correction, *p* = 0.0085, error bar: mean ± SD.

Under biofilm-forming conditions, the Δ*epsH* strain displayed significantly more biomass per CFU compared to WT biofilms at 2–14 days of culture (Fig. 6b, Supplementary Fig. 4). Statistical analysis using two-way ANOVA and Šídák’s multiple comparisons test revealed significant differences at 2- (p < 0.05), 4- (*p* < 0.0001), and 7-day-old (*p* < 0.005) biofilms. The high biomass per cell ratio reflects the significantly increased abundance of ECM in the Δ*epsH* biofilms. In planktonic culture, the biomass per bacterial cell was not significantly different in the Δ*epsH* and WT strains (unpaired t test, *p* = 0.0558, Fig. 6a).

Next, the macroscopic morphology of bacterial lawns was visualized on BHI Congo red plates^24^. Droplets of liquid cultures were incubated for 2 weeks (Supplementary Fig. 5 & Fig. 6c). On plates, the Δ*epsH* strain showed an even rougher colony morphology compared tothe the wild type strain. In other NTM species, the roughness of the colony morphology has been linked to lowered levels of glycopeptidolipids, high levels of trehalose mycolate and increased cording^25,26^.

To get more insight into the peculiar phenotype of the Δ*epsH* mutant, we evaluated the 3D morphology of 1-week-old submerged biofilms by confocal imaging of *M. marinum* Δ*epsH* and WT reporter strains expressing fluorescent tdTomato. The 3D imaging revealed that the Δ*epsH* mutant could form substantially larger cord-like bundles compared to the wild type strain (Fig. 6d, e). In addition, the total thickness of the biofilm measured at the 1-week time point was two-fold bigger in Δ*epsH* compared to wild type (p = 0.0085) (Fig. 6f). The observed alterations in the morphology of submerged biofilms of Δ*epsH* are in alignment with what would be expected from a mutant with more biofilm ECM.

### The level of eDNA and extracellular proteins, but not cellulose, is increased in *epsH*-deficient biofilms

**Fig. 7:**
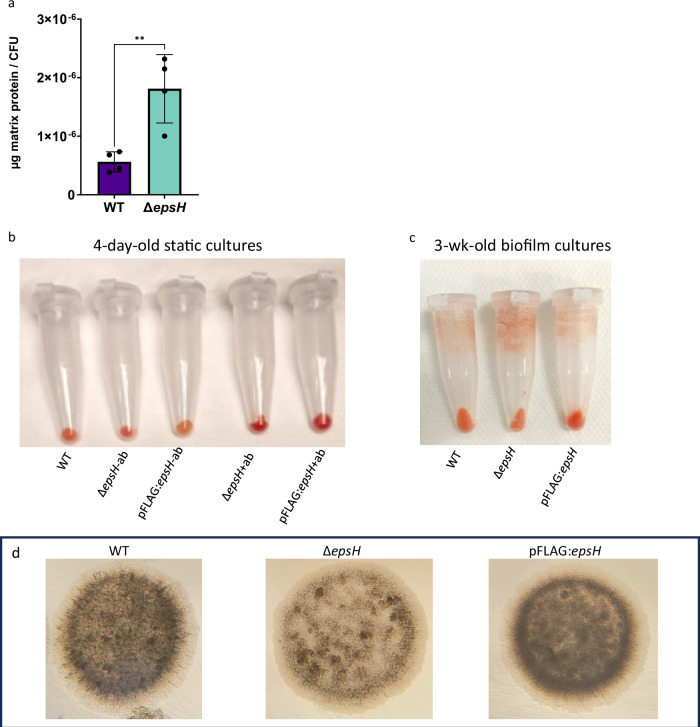
*epsH* deletion increases protein levels, but not cellulose, in biofilm-forming conditions and affects colony opacity. **a** *epsH* deletion results in elevated total protein levels in the ECM. Unpaired t test, *p* = 0.0064, error bar: mean ± SD. **b** Congo Red staining of 4-day old static cultures. Δ*epsH* and pFLAG:*epsH* were grown in the absence (−ab) and presence ( + ab) of strain specific antibiotics. Cultures were stained with 40 µg/ml Congo red for 2 h. **c** Congo Red staining of 3-week-old biofilm cultures. Cultures were stained with 40 µg/ml Congo red for 2 h. **d** Δ*epsH* strain forms less opaque colonies on agar. 6-day-old 5 µl inoculation spots.

To confirm the quantification of eDNA from the Δ*epsH* and WT biofilms with a non-staining-based method, a qPCR-crosslinker analysis was carried out. Δ*epsH* strain exhibited increased eDNA levels in biofilm and planktonic cultures (Supplementary Fig. 6). In biofilm formation, eDNA interacts with other ECM components, especially proteins and polysaccharides. These interactions have been shown to influence structural and functional properties of biofilms^27^. Next, the relative abundance of extracellular proteins and cellulose, the major polysaccharide component of mycobacterial biofilms, was measured in Δ*epsH* biofilm samples. Extracellular proteins were extracted from Δ*epsH* and WT biofilms by enzymatic shaving and quantified relative to the number of viable cells. In the Δ*epsH* mutant, extracellular protein levels were markedly elevated in one-week-old biofilms, with over 3-fold higher concentrations (p = 0.0064) compared to WT (Fig. 7a). However, based on Congo Red staining of Δ*epsH* and WT static planktonic cultures or 3-week-old biofilms, the level of cellulose was not altered in the epsH mutant biofilms (Fig. 7 b, c Supplementary Fig. 7). Interestingly, we noticed that including the selection antibiotic in the static planktonic cultures, the Δ*epsH* or the complemented strain, increased the Congo Red staining intensity (Fig. 7b and Supplementary Fig. 7). However, under this stress condition, there was no difference between the deletion and complemented strain suggesting that the deletion does not alter the cellulose level. Looking at the morphology of the colonies, it was observed that the Δ*epsH* colonies were less opaque on agar (Fig. 7d). In a study by Mushtaq et al. (2024), opaque *Acinetobacter baumannii* colonies were shown to have more extracellular polysaccharide moieties, which could suggest a deficiency in glycosyl moieties in the Δ*epsH* strain^28^.

These results suggest that *epsH* deletion increases the relative amount of eDNA and proteins in the ECM of mycobacterial biofilms, which is seen as a significant increase in the quantity of biofilm ECM. However, the reduced colony opacity may reflect a decrease in the abundance of sugar components within the ECM.

### Δ*epsH* biofilms are less tolerant to antibiotics in vitro

**Fig. 8:**
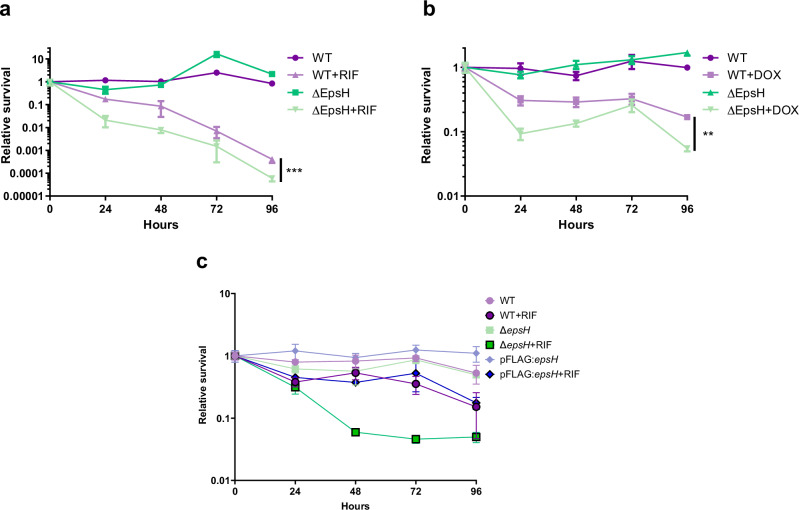
*epsH* deletion reduces antibiotic tolerance in biofilm forming conditions. 1-week-old microaerophilic, detergent-free liquid *M. marinum* biofilm cultures were exposed to high concentrations of antibiotics and surviving bacteria were plated daily to create time-kill curves for tolerance measurements**. a** The Δ*epsH* strain was less tolerant to 400 µg/ml of rifampicin (RIF) and **b** 250 µg/ml doxycycline (DOX). **c** Complementation of the *epsH*-deficient strain with *epsH*-expressing plasmid reverted the tolerance to wildtype levels.

Due to the increase in the relative amount of ECM in the Δ*epsH* strain, we hypothesized that this strain might be even more tolerant to antibiotics than the wild type strain. Liquid cultures of 1-week old Δ*epsH* and wildtype biofilms were exposed to extremely high doses of rifampicin (400 µg/ml) or doxycyclin (250 µg/ml) for 4 days. These two antibiotics are bacteriocidal against mycobacteria and have killing efficacy against biofilm forms within the timeframe of the experiment, when used at a high dose. Samples were plated on agar once a day to count the viable proportion of bacteria, and time-kill curves were drawn to measure antibiotic tolerance. Surprisingly, we found that despite the overall increased ECM, with significantly higher amounts of extracellular protein and DNA, the Δ*epsH* strain was significantly less tolerant to antibiotics than the wildtype (*P* < 0.001 with RIF and *P* < 0.01 with DOX) (Fig. 8). The MIC values of rifampicin and doxycyclin were not significantly altered in the Δ*epsH* strain suggesting no effects on antibiotic resistance (Supplementary Fig. 8).

### *M. marinum* lacking *epsH* does not show altered growth rate in the adult zebrafish model

**Fig. 9:**
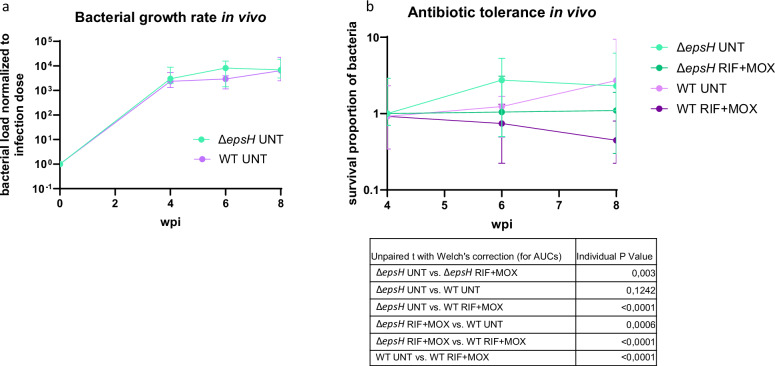
Δ*epsH* mutant strain demonstrates unaltered growth rate and increased drug tolerance in vivo. **a** Adult zebrafish were infected per intraperitoneal injection with 35 cfu of wt or 44 cfu of Δ*epsH M. marinum*. Bacterial loads were quantified at 4-, 6-, and 8-weeks post-infection (N = 16-24 per time point per group) by plating and counting colonies. The medians and 95% CI are shown. There was no difference in growth rate between the strains. **b** Adult zebrafish infected as in part a were treated with 32 mg/kg of rifampicin (RIF) and 15 mg/kg moxifloxacin (MOX) *per os* between 4 and 8 weeks of infection (N = 16–24 per group per time-point). The bacterial loads were quantified as in a and normalized to the median load at the beginning of the treatment. The medians and 95% CI are shown. The Δ*epsH* strain demonstrated significantly increased tolerance to antibiotics compared to the WT.

As biofilm formation is known to increase the virulence of *M. tuberculosis* in mice^3^ we wanted to see whether the Δ*epsH* mutant strain could differ from the wild-type in terms of growth rate in vivo. Adult zebrafish were infected with a low dose of either wild-type (35 ± 12 CFU per fish) or Δ*epsH* (44 ± 6 CFU) *M. marinum* (Fig. 9a), and the bacterial counts were measured by plating at four, six-, and eight-weeks post- infection. The difference in the initial infection dose was accounted for by normalizing all counts to the average infection dose. Despite the faster proliferation rate in in vitro biofilm culture and the more abundant ECM in biofilms in vitro, there was no increase in the growth rate for the Δ*epsH* mutant during infection.

### The lack of *epsH* increases antibiotic tolerance of *M. marinum* in the adult zebrafish

The ECM in biofilm is known to suppress the effects of antibiotics in vitro^16,29^ and in vivo^3^. Therefore, we were interested in looking at whether the in vivo efficacy of antibiotics would be lower against the Δ*epsH* mutant with more abundant ECM. Indeed, when adult zebrafish were treated with a combination of rifampicin and moxifloxacin between four and eight weeks of infection, the bacterial loads in zebrafish infected with the wildtype strain started slowly going down, but in the Δ*epsH* infection, the bacterial loads remained the same as at the beginning of the treatment (Fig. 9b). The area under the curve drawn using the starting-point normalized mean bacterial loads reveal that the AUC of the antibiotic-treated EpsH mutant was significantly larger than that of the antibiotic-treated WT group (*P* < 0.0001) suggesting a higher level of tolerance in the mutant. These results indicate that, unlike in the in vitro biofilm model, the absence of EpsH in vivo leads to even greater antibiotic tolerance than observed in wild-type *M. marinum* infections.

## Discussion

Biofilms contribute to antibiotic tolerance in mycobacteria by providing a protective environment that reduces the efficacy of treatment. Understanding the complex physiology of biofilms is essential for developing more effective and faster therapeutic strategies against mycobacterial infections. In this study, we have shown that deleting a putative glycosyltransferase *epsH* increases the relative amount of eDNA and leads to elevated overall ECM accumulation in *M. marinum* biofilms. The lack of *epsH* increased the antibiotic tolerance in vivo in the adult zebrafish model suggesting that the increased matrix provides additional protection against antibiotics during infection. This lays further ground for the possible utility of biofilm-matrix degradation as a viable treatment strategy for tuberculosis. Our results on this mutant also raise interesting speculations on the complex interplay between the different components of the ECM as well as the relative importance of the growth rate and the amount and composition of the ECM on antibiotic tolerance.

In our screen of a transposon-based mutant library, we used the amount of eDNA as a proxy for the level of biofilm formation, as eDNA is an essential component of the ECM of bacterial biofilms^11–14^. We identified several mutants with altered eDNA levels. Still, notably, the Δ*epsH* strain lacking a putative glycosyltransferase exhibited the most significantly elevated eDNA levels and higher biomass and extracellular protein/CFU. Generally, glycosyltransferases are known to enhance biofilm formation by generating or modifying exopolysaccharides^30,31^. Against this background, we were surprised to find a glycosyltransferase, the removal of which enhanced the overall biofilm formation. The observation that the lack of a glycosyltransferase in biofilms subsequently leads to a more significant accumulation of eDNA and extracellular protein in the extensive ECM suggests that EpsH may have a role in regulating the ECM of biofilms.

The annotation of QDR78_11175 carried out with PROKKA 1.11. by Das et al. 2018 (GenBank ID RFZ58776.1) suggests that this glycosyltransferase gene has a BcsA domain common in cellulose synthases, and is similar to *epsH* of *Bacillus subtilis*, where it acts as a crucial component in the β-(1 → 6)–linked poly-N-acetyl-D -glucosamine (PNAG) synthesis machinery affecting biofilm formation^30^. Both cellulose^3,10^ and PNAG^32^ have been detected in mycobacterial cultures, with cellulose specifically found in biofilms. Yet, the respective synthesis/secretion machineries of these polysaccharides remain uncharacterized in mycobacteria. In this study, we did not see a major difference in Congo red staining commonly used for visual detection of cellulose when the wild-type bacterial pellets were compared with Δ*epsH* pellets. This result suggested that the deletion of EpsH did not substantially decrease cellulose production. Based on the existing annotation GenBank ID RFZ58776.1 and the notion that mycobacteria contain PNAG^32^ it is likely that instead of cellulose, the EpsH may be involved in the PNAG synthesis of mycobacteria. Our result showing the lower opacity of the EpsH bacteria on agar also suggests that the ECM composition is altered in this strain in this strain. Mushtaq et al. 2024 reported *A. baumannii* variants with lower opacity contained less N-acetylglucosamine^28^. Looking at the PNAG staining in the work of Cywes-Bentley, it seems to be colocalized with the highest intensity DNA staining. This colocalization also suggests a possible interaction between PNAG and DNA in mycobacteria. The positive charge of PNAG^33^ would make it an amenable interaction partner of the negatively charged DNA. Based on the existing literature, we can speculate that the mycobacterial EpsH may be involved in PNAG synthesis and that PNAG and eDNA may also be linked in mycobacterial biofilms. Further investigation is needed to determine the precise mechanism by which *epsH* regulates ECM formation.

Looking into the literature of similar cases, we found that deleting a putative glycosyltransferase SMU_833 in S*treptococcus mutans* has decreased glucan and increased eDNA in biofilm^34^. In *Pseudomonas aeruginosa*, deleting a gene required for producing an exopolysaccharide alginate in biofilms also led to the increased release of eDNA into the ECM^35^. Hence, there are other examples where the increased release of eDNA and extracellular proteins compensates for the lack of an exopolysaccharide. It remains unclear whether the elevated eDNA levels in the Δ*epsH* mutant are due to increased cell lysis or active secretion since eDNA secretion can occur through various mechanisms. The most common mechanism is secretion through induced cell lysis under stressful conditions^12,16,27,36^. Still, a constant live/dead ratio of bacterial cells during eDNA accumulation indicates that eDNA can be actively delivered from living bacteria cells^14^. For *P. aeruginosa*, eDNA is released through cell death both through cell lysis and secretion of eDNA inside outer membrane vesicles^37^. In mycobacteria, Rose *et al*. (2016) have identified carbonic anhydrases among other proteins as active eDNA export regulators in *Mycobacterium avium* biofilms^38^. On the other hand, the sequence similarity of eDNA to genomic DNA in *M. tuberculosi*s and *M. intracellulare*, but specifically not in *M. avium*, indicates the origin of cell lysis in the first two species but possibly active transport in *M. avium*^16^. A transposon screen carried out in *M. avium* identified several mutants deficient in eDNA suggesting that this mycobacterial species possesses regulated mechanisms to release eDNA^38^. Taken together, the mechanisms of eDNA secretion may vary between mycobacterial species and have not been studied in *M. marinum*. In this study, most identified mutants had more eDNA than the wild type, and we were unable to reliably identify mutants with significantly less eDNA. However, as our screen was not saturated, we cannot make solid conclusions regarding the presence or absence of regulated eDNA release mechanisms in *M. marinum* biofilms. From our study on EpsH in *M. marinum*, we learned that the release of eDNA can be substantially increased by the deletion of a putative glycosyltransferase EpsH—a phenomenon also exemplified in other biofilm-forming species^34,35^.

Interestingly, the EpsH-deletion strain exhibited normal growth rate in planktonic cultures but significantly increased growth rate in the biofilm culturing conditions compared to WT or the complemented strain. It seems as if the product of the *epsH* gene in the wild-type context would act as a regulatory break to slow down bacterial growth in the biofilm-inducing conditions, whereas in its absence, the bacteria keep dividing at a fast pace, similar to that of planktonic bacteria. It can also simply be that there is a high fitness cost for the action of the EpsH in the biofilm conditions, when it allegedly becomes important in carrying out glycosylation of its targets. In future studies on this protein, it would be interesting to identify the exact targets of this protein in mycobacteria, to elucidate the mechanism in more detail.

The increased ECM in the Δ*epsH* biofilms is also likely to contribute to their increased tolerance to antibiotics, as biofilms, particularly those with thicker ECM, are known to be more tolerant to antimicrobial agents^39^. This increased antibiotic tolerance is significant in the context of TB, where the natural ability of *M. tuberculosis* to form biofilms can make it more difficult to treat. Indeed, when we measured the antibiotic tolerance of the Δ*epsH* strain to a combination of rifampicin and moxifloxacin in the adult zebrafish tuberculosis model, we saw that in the context of an infection, the mutant was more tolerant to antibiotics compared to wildtype. However, the antibiotic tolerance of the Δ*epsH* strain under biofilm-conditions in vitro, we were surprised to find that despite the increased ECM, the Δ*epsH* strain was substantially less tolerant to antibiotics compared to wildtype. Although the ECM is known to increase the antibiotic tolerance^39^, a fast growth rate has the opposite effect^40,41^. In vitro, in the biofilm medium, the Δ*epsH* strain was growing significantly faster compared to wild type both in terms of biomass as well as colony forming units. Therefore, it is plausible that the total level of tolerance is a composite of the protective effect of the ECM and the growth rate. In vitro, the increased growth rate of the Δ*epsH* may have reduced the tolerance to the extent that even the increased ECM was not protective. Alternatively, it may be that the lack of a glycosyl moiety transferred by EpsH, which is essential for antibiotic tolerance under in vitro conditions sensitizes the bacteria to antibiotics^28^. Under the in vivo conditions, on the other hand, there were no differences in the development of bacterial burdens between wild type and Δ*epsH* and the mutant strain with abundant ECM was more tolerant, as expected based on existing evidence on the protective role of increased ECM against antibiotics^16,42,43^.

The existing evidence supports the usefulness of DNase in treating biofilm infections by targeting extracellular DNA (eDNA), a key structural component of biofilms. Studies have shown that DNase can disrupt the biofilm matrix, leading to increased penetration of antibiotics and enhanced bacterial clearance. For instance, research demonstrated that the addition of DNase to antibiotic treatments significantly reduced biofilm biomass and the number of colony-forming units (CFUs) in both Gram-positive and Gram-negative bacterial biofilms^44–46^. In vitro evidence supports the utility of DNase treatment of *M. avium, M. chelonae* and *M. fortuitum* biofilms in combination with antibiotics^42,47^. Our results show that the Δ*epsH* strain with increased eDNA content is more tolerant to antibiotics than the wild-type providing indirect evidence that reducing eDNA may also be a viable strategy for treating mycobacterial infections in vivo. However, based on the knowledge from other bacterial species, it seems that extremely high concentrations of DNase are required for a substantial effect on antibiotic tolerance^46^. The fact that DNase has been approved for human pulmonary use to reduce the exacerbations of cystic fibrosis proves the safety of the administration of this enzyme^48^, making it an interesting option for treating mycobacterial biofilm infections in the future.

Overall, the study unveils the pivotal role of the glycosyltransferase EpsH in shaping mycobacterial biofilms. By deleting *epsH* in *M. marinum*, we discovered a dramatic increase in eDNA, extracellular protein, and total ECM accumulation. *M. marinum* lacking EpsH was fortified against antibiotics in vivo, supporting the connection between increased ECM and antibiotic tolerance. However, in vitro where the Δ*epsH* strain had a higher growth rate in biofilm conditions than the wild type, the increased ECM was not protective. The Δ*epsH* strain was less tolerant to antibiotics than the wild type. This intricate dance between ECM protection and growth rates underscores the complexity of biofilm biology. Based on our in vivo evidence in the zebrafish model, the potential of DNase to dismantle biofilms and boost antibiotic effectiveness is compelling. These insights pave the way for innovative treatments against mycobacterial infections, particularly tuberculosis, heralding a new era in the battle against these difficult-to-treat pathogens.

## Methods

### Bacterial strains and culture conditions

*Mycobacterium marinum* (ATCC 927) was cultured as described earlier^7^. Shortly, for planktonic cultures, an inoculum of *M. marinum* was transferred into a Middlebrook 7H9 medium supplemented with 10% (vol/vol) ADC (Fisher Scientific, NH, USA), 0.2% (vol/vol) glycerol, and 0.2% (vol/vol) Tween 80 (Sigma-Aldrich, MO, USA). Optical density (OD) was set to 0.05 in 600 nm wavelength, and the cells were cultured at 29 °C in bacterial culture tubes.

For the biofilm formation, a Middlebrook 7H9 medium with the ADC growth supplement but without Tween 80 or glycerol was used. The OD_600_ of an inoculum collected from agar plate preculture was set to 0.1, and the culture was sealed with a screw cap or parafilm M laboratory wrapping film and incubated at 29 °C for 2-14 days.

### Transposon library screen

*M. marinum* transposon library was created by φMycoMarT7 phage transductions. The phages contain a Himar1 transposon, a kanamycin resistance gene, and a transposase gene located outside the transposon boundaries to prevent several transposon insertions^49,50^. φMycoMarT7 phage stock is a gift from Eric Rubin Lab, Harvard.

The library was cultured on Middlebrook 7H10 plates with 10% (vol/vol) oleic albumin dextrose catalase (OADC) enrichment (Fisher Scientific, NH, USA), 0.5% (vol/vol) glycerol and 25 µg/ml kanamycin at 29 °C for 7–10 days in single colony density. Single colonies were collected and cultured in biofilm-forming conditions in 96-well plates at 192 µl/well for 12 days. Subsequently, 8 µl of 50 x GelRed (Biotium, Fremont, CA, US) was added to the culture for 36 hours. The fluorescence was measured in excitation of 280 nm and emission of 590 nm with EnVision 2104 Multilabel Plate Reader (PerkinElmer, Waltham, MA, US). The fluorescence signal was normalized to the OD_600_ signal of the culture.

### Validation of eDNA screen

To confirm the results from GelRed staining, the *eDNA high* and *eDNA low* hit clones were recultured in agar plates containing kanamycin (25 µg/ml) for 7 days. The clones were cultured in biofilm forming conditions on 96 well plates for 7 days. Next, the biofilms were homogenized by pipetting and stained with AccuBlue High Sensitivity dsDNA Quantitation Solution (Biotium, Fremont, CA, US) according to manufacturer’s instruction. The fluorescence signal was detected with EnVision 2104 Multilabel Plate Reader (PerkinElmer, Waltham, MA, US) and normalized to OD_600_ of the 7 days old biofilm cultures.

### Sequencing and data analysis

The genomic DNA was extracted with Quick-DNA Fungal/Bacterial Miniprep Kit (Zymo Research, California, USA). The sequencing was performed on NovaSeq PE150 system and the raw reads were quality filtered (Novogene, UK). The reads were assembled using SPAdes v3.14.1 with flag isolate and the resulting contigs were checked for the insertion of the phiMycoMarT7 (AF411123) transposon using BLASTn. Each of the mutants gave 1 hit to a complete transposon of phiMycoMarT7 (2068 nt). The surrounding regions (+/- 1000-2000 nt) were searched (discontiguous megablast program of BLASTn) against the reference genome *Mycobacterium marinum* ATTC 927 ^T^ (CP129323) and the disrupted regions were identical to genes with locus tags QDR78_11175 for the mutant A6E7, QDR78_00940 for the mutant A5A8 and QDR78_22550 in the mutant A3E5.

### DNase treatment

Biofilm cultures of WT and Δ*epsH* were established as explained above, and 12 µl DNase1 (Thermo Scientific, 1 U/ml) were added to 188 µl of biofilm culture at day 0. Individual samples were collected and stained with AccuBlue High Sensitivity dsDNA Quantitation Solution (Biotium, Fremont, CA, US) at given timepoints.

### Construction of complemented strain

To demonstrate that the observed deviations in the Δ*epsH* strain compared to the WT were explicitly caused by the loss of the *epsH* gene, a complementation strain was generated. The *epsH* gene was reintroduced following the methods described by Arnold et al. (2018)^51^. Specifically, the *epsH* gene was cloned into the pFLAG_attP vector and electroporated into the Δ*epsH* strain. To ensure plasmid retention, transformants were selected on 7H10 agar containing hygromycin (50 µg/mL). Complementation was validated using qPCR and AccuBlue assays, and the restored phenotypes were assessed under the same experimental conditions as the WT and Δ*epsH* strains.

### Quantitative PCR analysis

*M. marinum* WT, Δ*epsH* and pFLAG:*epsH* complementation strain were cultured in biofilm forming conditions for 7 days, after which the cells were collected by pelleting and homogenized in TRI reagent with glass bead mixture using FastPrep-24 5 G bead beating grinder and lysis system (MP Biomedicals, Santa Ana, CA, USA). The RNA was extracted according to the manufacturer’s instructions.

QPCR of the RNA samples was carried out with iTaq™ Universal SYBR® Green One-Step Kit (Bio-Rad, Hercules, CA, USA) according to manufacturer’s instructions. The cycling was performed with Bio-Rad C1000 Thermal Cycler (Bio-Rad) using the following parameters: reverse transcription at 50 °C for 10 min, initial denaturation at 95 °C for 1 min, followed by 35 cycles of denaturation at 95 °C for 10 s, and annealing/extension at 60 °C for 30 s. Fluorescence was measured at the end of each cycle to monitor the amplification of the target RNA. Melting curve analysis was performed after the final amplification cycle to verify the specificity of the PCR products. The data were analyzed using the comparative Ct (ΔΔCt) method, and the relative expression levels of the target genes were normalized to the housekeeping gene sigA. The primers used for *epsH* were (forward) 5’-GCATCTCATCATGGGGGAGG and (reverse) 5’-CACTGGAAATAGAGCCGCCA, and for *sigA* (forward) 5’- ATGGCGTTTCTGGACCTCAT and (reverse) AGCCCTTGGTGTAGTCGAAC. All samples were measured in triplicate.

### Planktonic growth analysis

To investigate the growth dynamics of WT, Δ*epsH* and pFLAG:*epsH Mycobacterium marinum* strains, three independent planktonic cultures were initiated for each strain in growth medium supplemented with Tween80. Cultures were standardized to an initial OD_600_ between 0.06 and 0.07. OD_600_ and CFU measurements were taken daily from each culture over a four-day period, following a 24-hour interval sampling schedule. CFU counts were recorded six days post-plating, and CFU/µL values were calculated to provide comparative growth curves for all strains. For plotting, the values were normalized to the values of the starting point.

### Biofilm growth analysis

To analyze the growth dynamics of WT, Δ*epsH*, and pFLAG:*epsH* strains under biofilm-forming conditions, three cultures of each strain were initiated in growth medium and standardized to an initial OD600 of 0.1. CFU measurements were taken daily over a seven-day period, with samples collected at 24-hour intervals. CFU counts were recorded six days after plating, and CFU/µL values were calculated to generate comparative growth curves. For visualization, the values were normalized to the starting point.

### Biomass analysis

*M. marinum* WT, Δ*epsH*, and pFLAG:*epsH* complementation strain were cultured in biofilm-forming conditions for 14 days (50 ml per sample, *n* = 4). Samples were collected by pelleting and the pellet was suspended in 20 mL of Middlebrook 7H9 medium supplemented with 10% (vol/vol) ADC (Fisher Scientific, NH, USA), 0.2% (vol/vol) glycerol, and 0.5% (vol/vol) Tween 80 (Sigma-Aldrich, MO, USA). After 2 h of incubation at +29 °C, 400 µl samples were collected into separate tubes, and a viable plate count was performed after 7 days of incubation. The remainder of the samples were pelleted in pre-weighed Eppendorf tubes, after which the supernatants were carefully removed. Biomass was then weighed, and the relative biomass per cell was calculated.

### Quantification of extracellular proteins

One-week-old biofilms of *M. marinum* WT and Δ*epsH* mutant were generated in 10 mL of Middlebrook 7H9 with 10% ADC supplement at +29 °C under microaerophilic conditions. One mL of the dispersed biofilm cultures was used for measuring CFUs (cells were pelleted, suspended, and shaken in PBS with 0.4% Tween 80 for 1 h prior to serial dilutions from -1 to -8 on MiddleBrook 7H10 agar with 10% OADC and 0.5% glycerol). For measuring the protein/peptide concentrations, the biofilm cell cultures (9 mL) were pelleted and washed once with 50 mM sodium acetate (pH 5.2) and then suspended in 50 mM TEAB (pH 8.5). Extracellular proteins were released by enzymatic treatment with trypsin at a final concentration of 42 ng/µL ( + 37 °C for 30 min). The released polypeptide concentrations were determined at A_205_ using NanoDrop. The experiment was repeated with four independent replica biofilm samples for both the WT and Δ*epsH* cells.

### Congo red staining

*M. marinum* WT, Δ*epsH* and pFLAG:*epsH* complementation strain were cultured in 10 ml of growth medium supplemented with Tween-80 for 4 days, or in biofilm-forming conditions for 3 weeks. For the 4-day-old cultures, the mutant strain Δ*epsH* and complementation strain pFLAG:*epsH* were grown with and without strain specific antibiotics (kanamycin (50 g/µl), or kanamycin and apramycin (50 µg/ml), respectively). Antibiotics were added to the biofilm cultures in the first day of the 3-week period. The cultures were collected by centrifugation (5000 x g 5 min). The pellets were suspended in 0.05 M citrate buffer and incubated with or without 5 mg/ml cellulose degrading enzyme (Cellulase from *Tricohoderma sp*, C1794 Sigma) at 37 °C o/n. The cells were washed once with sterile water before adding 1 ml of 40 µg/ml Congo red solution (Congo red (C.I. 22120), 1.01340 Merck). The cells were incubated at 28 °C 150 rpm for 2 h and washed three times with PBS. The staining was inspected visually.

To visualize the morphology of *M. marinum* WT, Δ*epsH* and pFLAG:*epsH*, the strains were grown on Congo red agar modified based on the instruction of the commercial Congo red provider BioGnost (52 g/l Brain Heart Infusion (BHI) agar (70138 Millipore) supplemented with 10% OADC and 0.08% Congo red). WT and Δ*epsH* strains were streaked on agar from glycerol stocks and grown at 28 °C for 3 weeks. WT, Δ*epsH* and pFLAG:*epsH* were also made into suspensions (OD_600_ = 0.1), and 5 µl spots of the suspension were pipetted on agar and grown at 28 °C for 2 weeks.

### In vitro time-kill curves

*M. marinum* WT, Δ*epsH* and pFLAG:*epsH* complementation strain were cultured in 96 well plates in biofilm forming conditions for 7 days. Subsequently, cultures were exposed to rifampicin (400 ul/ml) or doxycycline (250 ug/ml). Before adding antibiotics and at various times after, cells were harvested to a Middlebrook 7H9 medium supplemented with 10% (vol/vol) ADC (Fisher Scientific, NH, USA), 0.2% (vol/vol) glycerol, and 0.5% (vol/vol) Tween 80 (Sigma-Aldrich, MO, USA) and incubated for 2 h at 29 °C in gentle agitation. Samples were plated to Middlebrook 7H10 plates with 10% (vol/vol) oleic albumin dextrose catalase (OADC) enrichment (Fisher Scientific, NH, USA) and 0.5% (vol/vol) glycerol, and cell viabilities were determined by counting the number CFUs 7 days post plating.

### MIC assessment

The MICs of rifampicin and doxycycline against *M. marinum* wild type, Δ*epsH*, and pFLAG:*epsH* strains was determined using a broth microdilution assay in a 96-well plate format. Serial two-fold dilutions of the antibiotics were prepared in Middlebrook 7H9 medium with 10% OADC enrichment, covering a concentration range of 0.06-2 µg/ml for rifampicin and 2-64 µg/ml for doxycycline. Bacterial cultures were adjusted to OD_600_ of 0.1 and inoculated into the wells. The plates were incubated at 29°C for 7 days and bacterial growth was visually assessed by comparing wells to a negative control (medium only). The MIC was defined as the lowest antibiotic concentration at which no visible bacterial growth was observed.

### Zebrafish maintenance and ethics statement

A total of 4–12 months old male AB wildtype zebrafish (Danio rerio) obtained from Tampere Zebrafish Core Facility were used for in vivo experiments. Fish were housed in a flow-through system with a 14/10 h light/dark cycle and fed daily with GEMMA Micro 500 (Skretting, USA), mixed with gelatin and antibiotics for treatment groups. Water conditions were maintained at pH 7.6, conductivity 800 µS, and temperature 28 °C, adjusted with sea salt and NaHCO3. Fish were housed in groups of 10–25. The zebrafish maintenance and experiments were in accordance with the Animal Experiment Board in Finland (the licenses ESAVI/10079/04, ESAVI/17803/2019 and ESAVI/14286/2022) and were carried out in accordance with the EU-directive 2010/63/EU on the protection of animals used for scientific purposes and with the Finnish Act on Animal Experimentation (62/2006).

### Zebrafish infections

An inoculum of *M. marinum* ATCC 927 (wildtype) or *M. marinum* Δ*epsH* was transferred into a Middlebrook 7H9 medium supplemented with Tween 80 and cultured for 4 days at 28 °C. The bacteria culture was diluted to OD_600_ of 0.07-0.09 and cultured for an additional 2 days to approximately an OD_600_ of 0.5. 1 ml of culture was centrifuged for 3 minutes at 10,000 x g. The supernatant was discarded, and the pellet resuspended in 1 ml of 1x PBS. Bacteria suspension was diluted according to the predetermined OD_600_ vs. CFU/µl curve aiming to reach 7 CFU/µl concentration by using 1x PBS with 0.3 mg/mL phenol red (Sigma-Aldrich, St. Louis, MO, USA). The suspension was pulled three times through a 27 G needle (Henke-Sass Wolf, Tuttlingen, Germany) with a 1 ml syringe (Terumo, Tokyo, Japan). The fish were anesthetized by brief (up to 90 s) immersion in water containing 0.02% of the most commonly used fish anesthetic, 3-aminobenzoic acid ethyl ester (Sigma-Aldrich, St. Louis, MO, USA) until the fish became sedated and unresponsive to touch. The anesthetized fish were intraperitoneally injected with 5 µl using an Omnican 100 30 G insulin needle (Braun, Melsungen, Germany) and immediately returned into a freshwater recovery tank. Multiple samples were plated into 7H10 plates during infection to verify the infection dose. After *M. marinum* infection, humane endpoint criteria approved by the national ethical board were followed. Fish with any of the following symptoms: lack of response to touch, abnormal swimming, gasping, observable swelling, observable wasting, or loss of scales, were euthanized by prolonged (10–15 min) immersion into water containing an overdose (0.04%) of 3-aminobenzoic acid ethyl ester, until the breathing and heartbeat stopped.

For sample collection, the fish were euthanized as above. The internal organs of euthanized zebrafish were collected into 1.5 ml homogenization tubes (OMNI International, Kennesaw, GA, USA) containing six 2.8 mm ceramic beads (OMNI International, Kennesaw, GA, USA) and 100 µl 1x PBS. 300 µl of 6.66% SDS in 1x PBS was added to the samples and the organs were homogenized using FastPrep-24 5 G at 6.5 m/s for 2×40-second cycles with a 60-second pause. Samples were transferred to 2 ml centrifuge tubes, mixed 1:1 with MycoPrep reagent (Becton, Dickinson and Company, NJ, USA), and incubated for 15 min with occasional inversion After adding 1 ml of MycoPrep phosphate buffer, the samples were centrifuged at 3,000 × g for 20 minutes. The supernatant was discarded, and the pellet was resuspended in 1 ml of 1x PBS. Tenfold serial dilution up to 10-7 was made to 1x PBS using 96-well plates and 5 µl droplets were plated as triplicates on 7H10 agar plates. Colonies were counted after 6 days of incubation at 28 °C.

### Microscopy

To enable fluorescence-based visualization, both wildtype and Δ*epsH Mycobacterium marinum* strains were transformed with the tdTomato-expressing pTEC27 vector. pTEC27 was a gift from Lalita Ramakrishnan (Addgene plasmid no. 30182). This allowed visualization of biofilms and assessment of structural differences between strains. Biofilms of tdTomato expressing strains of WT and Δ*epsH* were cultured for 7 days in biofilm-forming conditions described earlier^7^. Imaging was performed using a Nikon A1R+ confocal laser scanning microscope equipped with a Nikon Apo LWD 40x WI λS DIC N2 objective (1024 ×1024 resolution, 310 nm pixel size, and 325 nm z-step, water immersion). Laser power was set to 0.1% of full power, and detector sensitivity/gain set to 10. Depth color coding and maximum intensity projections were processed using Fiji-ImageJ software. Prior to biofilm thickness analysis, slices with a mean intensity below 15 were excluded from the top and bottom of the biofilm to remove areas with minimal signal. The number of remaining slices was then multiplied by the step size (0.325 µm) to calculate the total biofilm thickness.

### Crosslinker analysis

Biomass of one-week-old biofilms of *M. marinum* WT and Δ*epsH* mutant (10 ml per sample, *n* = 4) were collected by pelleting, resuspended to 1 ml of medium, and diluted 1:100 to PBS. Samples were taken for plating and further CFU counting. Subsequently, each sample was separated to two Eppendorf tubes, and 1:100 volume of 1 µM PCR crosslinker (Currently available as Viability PCR Reagent Kit A8881 or A8883 from Promega) was added to half of the samples. Samples were incubated at +37 °C for 1 h, mixing once during incubation. Twenty µl of neutralization buffer per sample was added, followed by 15 min incubation at RT. For qPCR analyses, primers targeting ITS area of *M. marinum* were designed to amplify 536 bp long sequence (GCACCTCACCGCTATTGGTT and GCCCTCTCCAACTCCAACAA). Total DNA from biofilm samples +/- crosslinker was extracted by Blood & Tissue Kit (Qiagen).

### Data analysis, graphs and statistics

Data were analysed using Microsoft Excel and GraphPad Prism 10. All graphs were created using Prism 10. Statistical significance was assessed with the appropriate test according to the methods described in each figure caption. The alpha level used in this study was 0.05. The graphical abstract was created using BioRender.

## Supplementary information

Supplementary_Figures

## Data Availability

The datasets used and/or analysed during the current study available from the corresponding author on reasonable request.
