# Supplementary material for: M.marinum lacking epsH shows increased biofilm formation in vitro and boosted antibiotic tolerance in zebrafish
Source: NPJ Biofilms Microbiomes. 2025 Jun 14;11:109. doi: 10.1038/s41522-025-00743-5 (PMC12167362; doi:10.1038/s41522-025-00743-5)
Supplement: Supplementary file 1 — Supplementary_Figures [file 41522_2025_743_MOESM1_ESM.pdf]

Supplements

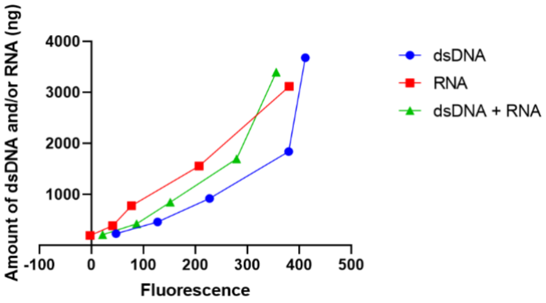

Suppl. Fig. 1. Affinity of GelRed® for different nucleic acids. Gelred stains both DNA and RNA.

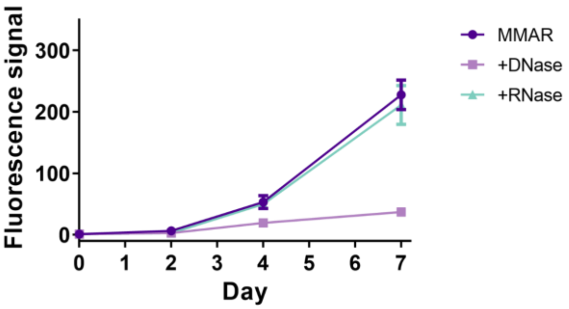

Suppl. Fig. 2. RNase treatment does not affect AccuBlue dye based eDNA detection, which indicates the specificity of AccuBlue dye to DNA.

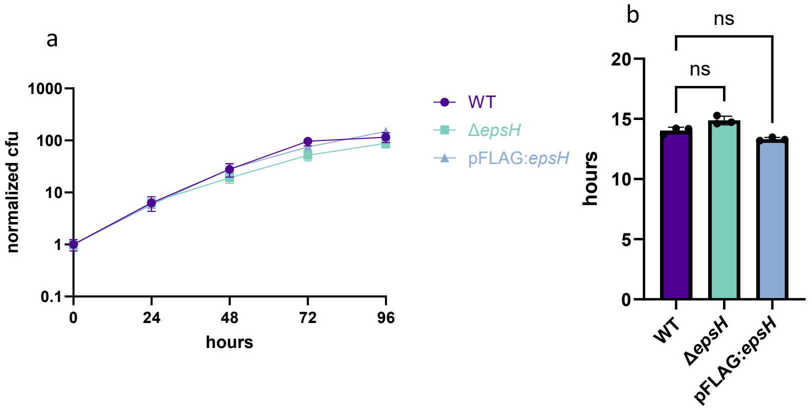

Suppl. Fig. 3. *epsH* deletion doesn't affect growth in planktonic conditions. **a** The growth curve of Δ*epsH* strain was similar to the growth curve of WT strain and **b** there was no significant differences in the generation times of the strains. Brown-Forsythe and Welch ANOVA followed by Dunnett's T3 multiple comparisons test. p>0.05, error bar: mean ± SD.

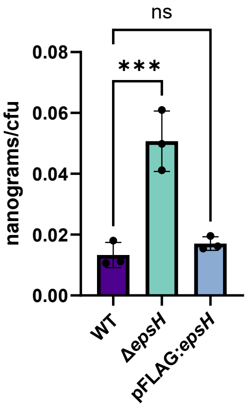

Suppl. Fig. 4. *epsH* deletion increases biomass per cell. Biomass per cell was significantly increased in 14-day-old Δ*epsH* biofilms. Complementation of the *epsH*-deficient strain with *epsH*-expressing plasmid reverted the biomass per cell to wild type levels. One-way ANOVA followed by Dunnett's multiple comparisons test. ***p=0.0006, error bar: mean ± SD.

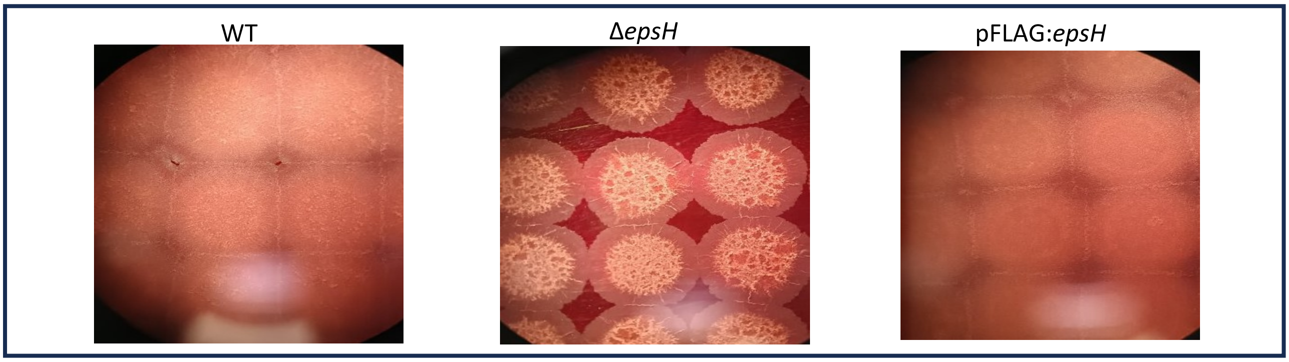

Suppl. Fig. 5. Growth patterns of WT, Δ*epsH* and pFLAG:*epsH M. marinum* strain on Congo red agar after 2 weeks. Images depict 5 µL inoculation spots. WT colonies exhibit smoother surface, while Δ*epsH* colonies display rougher surface. pFLAG:*epsH* strain shows growth patterns resembling WT.

Supplements

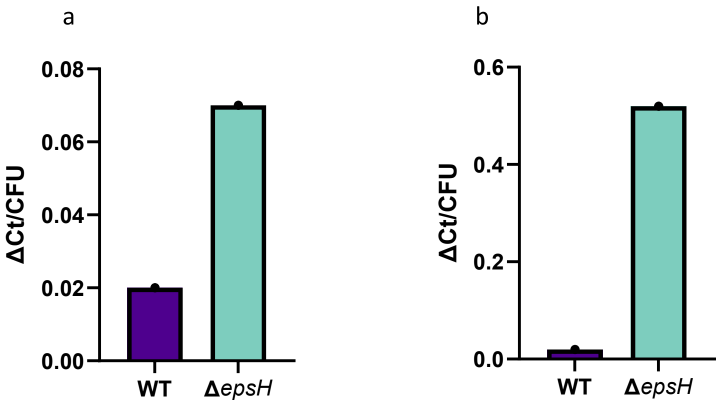

Suppl. Fig. 6. Amount of eDNA in Δ*epsH* and wild type strains was confirmed with crosslinker-qPCR assay, where crosslinker binds to eDNA and prevents qPCR amplification. The ΔCt value of samples with and without crosslinker will indicate the amount of eDNA in the sample. ΔCt values were normalized to CFU counts. Higher eDNA levels for Δ*epsH* strain in comparison to WT were detected in both **a** planktonic (3 days old, in logarithmic growth phase) and **b** biofilm (7 days old) samples.

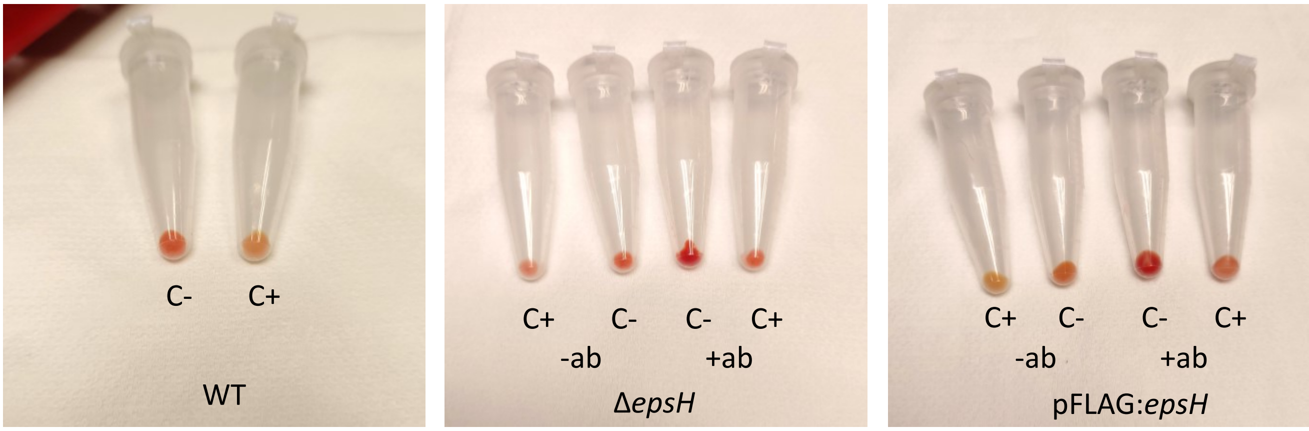

Suppl. Fig. 7. Comparison of cellulase-treated and non-treated WT, ΔEpsH and pFLAG:EpsH cultures after Congo Red staining. Cellulase treatment (C+) decreased staining compared to non-treated samples (C-) (visual inspection). 4-day-old static cultures were treated with 5 mg/ml cellulase o/n at 37 ° C or incubated at same conditions without the enzyme. Cultures were then stained with 40 µg/ml Congo red for 2 h. Δ*epsH* and pFLAG:*epsH* were grown in the absence (-ab) and presence (+ab) of strain specific antibiotics.

	**WT**	**Δ*epsH***	**pFLAG:*epsH***
RIF	2 µg/ml	2 µg/ml	1 µg/ml
DOX	32 µg/ml	16 µg/ml	16 µg/ml

Suppl. Fig. 8. Minimum inhibitory concentration (MIC) of rifampicin (RIF) and doxycycline (DOX) for *Mycobacterium marinum* shows no major differences between strains. MIC values were determined using a broth microdilution assay for the WT, Δ*epsH*, and pFLAG:*epsH* strain.
